# Assessing the Power of the Francis Psychological Type and Emotional Temperament Scales (FPTETS) to Predict Professional Burnout alongside Lifestyle and Support Choices Among Catholic Priests in Portugal

**DOI:** 10.1007/s10943-025-02319-1

**Published:** 2025-05-20

**Authors:** Janaína Mengal Gomes Fabri, Leslie J. Francis, Ursula McKenna, Liliana Isabel Faria Roldão, Sílvia Caldeira, Eliane Ramos Pereira

**Affiliations:** 1 Faculty of Nursing, State University of Rio de Janeiro, Rio de Janeiro, Brazil; 2 Health Care Sciences at the Fluminense Federal University, Rio de Janeiro, Brazil; 3 Health Sciences Institute, Portuguese Catholic University, Lisbon, Portugal; 4 Centre for Educational Development, Appraisal and Research (CEDAR), University of Warwick, Coventry, UK; 5 World Religions and Education Research Unit, Bishop Grosseteste University, Lincoln, UK

**Keywords:** Francis Burnout Inventory, Psychological type, Emotional stability, Extraversion, Leisure activities, Clergy studies

## Abstract

This study was designed to test the power of personality, engagement with leisure activities, and professional support, in order to predict susceptibility to professional burnout among Catholic priests in Portugal. Data provided by 208 priests who completed both the Francis Psychological Type and Emotional Temperament Scales and the Francis Burnout Inventory demonstrated that reported levels of burnout were significantly lower among stable extraverts and among those who engaged more frequently with leisure activities, while no further predictive power was associated with engaging a discipler or mentor. These findings carry implications for the pastoral care and pastoral oversight of priests.

## Introduction

A central question in understanding (and hence mitigating) levels of professional burnout among religious professionals concerns evaluating the comparative predictive power of internal and contextual factors. Internal factors may include personal differences (e.g. age, ethnicity, and within some denominations sex) and psychological differences (e.g. personality and psychopathology), while contextual factors may include institutional and structural factors. Lifestyle choices and professional support choices may also play a role. A particular focus of research in this area has been the role of individual differences in personality in predicting susceptibility to professional burnout among religious professionals, with studies highlighting the predictive capability of personality over contextual or situational factors. This body of research has employed the big five factors model of personality proposed by Costa and McCrae (1985) and the three major dimensions model of personality proposed by Eysenck and Eysenck (1975, 1991).

The big five factors model operationalises the factors of extraversion, neuroticism, agreeableness, openness, and conscientiousness. Studies employing this model alongside the Maslach Burnout Inventory (Maslach & Jackson, 1986) have been reported by Miner (2007a, 2007b), Joseph et al. (2011), and Stephens (2020). For example, in a study among 603 Protestant clergy in the USA, Stephens (2020) found that: depersonalisation was positively associated with neuroticism (*β* = .45), negatively associated with extraversion (*β* = −.07), negatively associated with agreeableness (*β* = −.27), and independent of openness and conscientiousness; emotional exhaustion was positively associated with neuroticism (*β* = .60), positively associated with conscientiousness (*β* = .07), negatively associated with extraversion (*β* = −.22), and independent of openness and agreeableness; and personal accomplishment was negatively associated with neuroticism (*β*=−.23), positively associated with openness (*β* = .24), positively associated with conscientiousness (*β* = .18), positively associated with extraversion (*β* = .28), and independent of agreeableness.

The three major dimensions model operationalises the dimensions of extraversion, neuroticism, and psychoticism. Studies employing this model alongside the Maslach Burnout Inventory (Maslach & Jackson, 1986) have been reported by Kay (2000), Rutledge and Francis (2004), Francis, Louden, and Rutledge (2004), and Francis and Turton (2004a, 2004b). For example, in a study of 1,071 Anglican clergymen in England, Rutledge and Francis (2004) found that: depersonalisation was positively associated with neuroticism (*β* = .32), positively associated with psychoticism (*β* = .24), and negatively associated with extraversion (*β* = −.14); emotional exhaustion was positively associated with neuroticism (*β* = .46), positively associated with psychoticism (*β* = .13), and negatively associated with extraversion (*β* = −.15); and personal accomplishment was positively associated with extraversion (*β* = .42), negatively associated with neuroticism (*β* = −.22), and negatively associated with psychoticism (*β* = −.06).

More recently, Crea et al. (2024) have tested the power of the HEXACO model of personality proposed by Lee and Ashton (2012) alongside the Francis Burnout Inventory (Francis, Kaldor, et al., 2005). The HEXACO model purports to operationalise the five factors established by the big five factor model with the addition of a sixth factor styled as honesty and humility. This study found that satisfaction in ministry was positively associated with extraversion (*β* = .32) and conscientiousness (*β* = .23), while emotional exhaustion in ministry was negatively associated with extraversion (*β* = −.25) and conscientiousness (*β* = −.23), *and* negatively associated with honesty and humility (*β* = −.33).

### Introducing Psychological Type Theory

Alongside the major three dimensions of personality (Eysenck & Eysenck, 1975, 1991) and the big five factors of personality (Costa & McCrae, 1985), psychological type has gained visibility within the psychology of religion more generally (see Lewis, 2012, 2015, 2021a, 2021b, 2022; Village, 2011) and within clergy studies in particular. Introduced to clergy studies initially by several small studies (Greenfield, 1969; Harbaugh, 1984; Holsworth, 1984), Macdaid et al. (1986) assembled the profiles of 15 different religious groups, including the profile of 1,554 Protestant ministers and the profile of 1,298 Catholic priests. More recently, a series of studies conducted in the UK have compiled the profile of Apostolic network leaders, Baptist ministers, Church in Wales clergy, Church of England clergy, Methodist ministers, lead elders within the Newfrontiers network of churches, Roman Catholic priests, and Salvation Army officers (for overview see Francis, Haley, & McKenna, 2023).

Advocates for employing psychological type theory within clergy studies suggest on theological grounds that the major three dimensions model and the big five factor model fail to distinguish between individual differences in personality, character, and psychopathology (see Francis & Village, 2015; Francis, Fawcett, & McKenna, 2023). Indeed, the Eysenckian major three dimensions model explicitly hypothesises continuity between normal personality and neurotic disorder and psychotic disorder in the two dimensions named as neuroticism and psychoticism (see especially Eysenck & Eysenck, 1976). The big five factor model valorises qualities of openness, agreeableness, and conscientiousness against the opposite poles of these factors (see Lloyd, 2015). Within a theologically informed framework that conceptualises human beings created in the image of God (doctrine of creation), tarnished by original sin (doctrine of fall), and restored in Christ (doctrine of salvation), it becomes important to disentangle these strands of personality (reflecting the divine image), character, and psychopathology (see Francis & Village, 2015).

Critics of psychological type theory focus on the theoretical roots for the theory and on the unsatisfactory psychometric properties of the core measure of psychological type theory, the Myers–Briggs Type Indicator (Myers & McCaulley, 1985) as not complying with standard psychometric practice (see further Francis, 2005; Lloyd, 2008, 2024). The Francis Psychological Type Scales were designed to meet standard psychometric practice in operationalising the constructs defined by psychological type theory (see Francis, Laycock, & Brewster, 2017; Francis & Village, 2022; Payne et al., 2021; Village, 2021).

The core of psychological type theory distinguishes between two cognitive processes: the perceiving process and the judging process. These two processes are then contextualised within a theory of psychological energy (orientation) and a theory concerning the way in which the outside world is approached (attitude). In psychological type theory the perceiving process, the judging process, the orientation, and the attitude towards the outside world are each characterised by two contrasting poles or types.

The perceiving process is concerned with identifying ways in which individuals take in information. Jung (1971) describes this as the irrational process because it is not concerned with data evaluation, but simply with data gathering. Here the two types are defined as sensing and intuition. Sensing types focus on the present realities: they are practical people. Intuitive types focus on the future potentialities: they are visionary people.

The judging process is concerned with identifying ways in which individuals evaluate information. Precisely for this reason, Jung (1971) describes this as the rational process because it is actively concerned with data evaluation and decision-making. Here the two types are defined as thinking and feeling. Thinking types are concerned with objective analysis and dispassionate logic. They are concerned with the good running of systems and organisations and put such strategic issues first. Feeling types are concerned with subjective evaluation and personal involvement. They are concerned with good relationships between people and put such interpersonal issues first.

The orientations are concerned with identifying the source of psychological energy. Here the two types are defined as extraverts and introverts. Extravert types are energised in the outer world of people and things. They are exhausted by long periods of silence and solitude. They need to re-energise through the stimulation that they receive from people and places. Introvert types are energised by the inner world of ideas and reflection. They are exhausted by long periods of social engagements and activities. They need to re-energise through the stimulation they receive from their own company and tranquillity.

The attitudes towards the outer world are concerned with identifying which of the two processes (perceiving or judging) individuals prefer to exercise in the outer world. Here the two types are defined as perceiving and judging. Perceiving types employ their preferred perceiving function (sensing or intuition) in the outer world. Because their outer world is where the irrational, data gathering function is employed, perceiving types appear to others to be laid-back, flexible, spontaneous people. Judging types employ their preferred judging function (thinking or feeling) in the outer world. Because their outer world is where the rational, data evaluating function is employed, judging types appear to others to be well-organised, decisive, and prepared people.

The Francis Psychological Type Scales assess preferences for extraversion, introversion, sensing, intuition, thinking, feeling, judging, and perceiving by identifying ten clear characteristics associated with each preference and by pairing such characteristics in forced-choice format against the opposite preference. The resulting eight scale scores are then weighted to transform continuous scale scores into categorical preferences.

In the wider research literature concerning psychological type, some evidence has been produced linking psychological type with individual differences in work-related psychological health. For example, Reid (1999) reviewed a series of four unpublished doctoral dissertations and one published study which had assessed the relationship between psychological type and scores recorded on the Maslach Burnout Inventory (Maslach & Jackson, 1986). The stable finding across four of these five studies was that individuals with a preference for extraversion appeared to be less prone to burnout than people with a preference for introversion. More detailed findings reported by Lemkau et al. (1988) noted that extraverts recorded significantly higher scores on personal accomplishment than introverts, that thinking types recorded significantly higher scores on depersonalisation than feeling types, and that judging types recorded significantly higher scores on emotional exhaustion than perceiving types. Findings reported by Myers et al., (1998, p. 238) noted that introverts recorded significantly higher scores than extraverts on emotional exhaustion and on depersonalisation.

Employing the Francis Burnout Inventory (Francis, Kaldor, et al., 2005), a series of nine studies have explored the connection between the two measures of emotional exhaustion in ministry and satisfaction in ministry and the four components of psychological type (the two orientations, the two perceiving functions, the two judging functions, and the two attitudes) as assessed by the Francis Psychological Type Scales (Francis, 2005). These studies have been conducted among 748 clergy serving in the Presbyterian Church (USA) by Francis, Wulff, and Robbins (2008), among 3,715 clergy from Australia, England, and New Zealand by Francis, Robbins, et al. (2009), among 521 clergy serving in rural ministry in the Church of England by Brewster, Francis, and Robbins (2011), among 874 clergywomen serving in the Church of England by Robbins and Francis (2010), among 134 lead elders within the Newfrontiers network of churches serving in the UK by Francis, Gubb, and Robbins (2012), among 212 Australian clergywomen drawn from 14 denominations or streams of churches by Robbins et al. (2012), among 266 clergymen serving in the Church in Wales by Francis, Payne, and Robbins (2013), among 155 Catholic priests serving in Italy by Francis and Crea (2015), and among 589 Canadian Baptist clergy by Durkee-Lloyd (2016).

In terms of emotional exhaustion, all nine studies reported significantly higher scores recorded by introverts than by extraverts. Five of the nine studies also reported significantly higher scores recorded by thinking types than by feeling types. In terms of satisfaction in ministry, eight of the nine studies reported significantly higher scores recorded by extraverts than by introverts. Five of the nine studies also reported significantly higher scores recorded by feeling types than by thinking types. The clear message from these findings is that extraverts and feeling types fare better in ministry than introverts and thinking types.

### The Francis Psychological Type and Emotional Temperament Scales

Although psychological type theory has its origins within a very different conceptual framework from other well-established and widely accepted models of personality developed within the individual differences tradition, several studies have explored the connections between measures of psychological type theory (employing the continuous scale scores underpinning type categorisation) and the scales proposed by other models. For example, the connection between scores recorded on the Myers–Briggs Type Indicator (Myers & McCaulley, 1985) and various editions of the Eysenckian personality measures (Eysenck & Eysenck, 1964, 1975; Eysenck et al., 1985) have been reported by Wakefield et al. (1976), Steele and Kelly (1976), Campbell and Heller (1987), Sipps and Alexander (1987), Landrum (1992), Saggino and Kline (1995), Francis and Jones (2000), Furnham et al. (2001), and Francis, Craig, and Robbins (2007). These studies tend to find that the MBTI measures of introversion and extraversion are correlated with the Eysenckian extraversion scale, and the MBTI measures of judging and perceiving are correlated with the Eysenckian psychoticism scale.

The connection between scores recorded on the Myers–Briggs Type Indicator and various measures of the Big Five Factor model of personality have been reported by McCrae and Costa (1989), MacDonald et al. (1994), Furnham (1996), Parker and Stumpf (1998), Furnham et al. (2001), Furnham et al. (2003), and Renner et al. (2014). These studies tend to find that the MBTI measures of introversion and extraversion are correlated with the Big Five extraversion scale; that the MBTI measures of judging and perceiving are correlated with the Big Five conscientiousness scale; and that the MBTI measures of sensing and intuition are correlated with the Big Five openness to experience scale.

These studies that set measures of psychological type theory alongside other well-established and widely accepted models of personality developed within the individual differences tradition consistently draw attention to the absence of a measure of emotionality within the framework of psychological type theory. It is for this reason that Village and Francis (2023) introduced a fifth measure, a measure of emotionality, alongside the four established measures within the Francis Psychological Type Scales (FPTS), leading to the Francis Psychological Type and Emotional Temperament Scales (FPTETS).

While the Francis Psychological Type and Emotional Temperament Scales were developed to assign individuals to type categories, the underlying continuous scale scores have also proved to be fruitful in correlational and regression analyses. With that usage in mind, Village and Francis (2024) proposed a short version, comprising five six-item scales.

### Assessing Burnout Among Clergy

Recent studies assessing burnout among clergy have tended to employ either the Maslach Burnout Inventory (FBI; Maslach & Jackson, 1986) or the Francis Burnout Inventory (FBI; Francis, Kaldor, et al., 2005). The MBI assesses three components, emotional exhaustion, depersonalisation, and lack of personal accomplishment, and proposes a sequential model whereby emotional exhaustion leads to depersonalisation and depersonalisation leads to lack of personal accomplishment (Maslach, 2003). The FBI assesses two components, emotional exhaustion and satisfaction in ministry, and proposes a balanced affect model whereby satisfaction in ministry mitigates the deleterious effects of emotional exhaustion. A series of studies have validated the theory of balanced effect on relevant outcome measures, including frequency of thoughts of leaving ministry. These validation studies include work reported by Francis, Village, et al. (2011) among 744 clergy in The Presbyterian Church USA, by Francis, Laycock, and Brewster (2017) among 658 clergy in the Church of England, by Francis, Laycock, and Crea (2017) among 155 priests in the Roman Catholic Church in Italy, by Francis, Crea, and Laycock (2017) among 95 priests and 61 sisters in the Roman Catholic Church in Italy, by Village et al. (2018) among 358 Anglican clergy in the Church of Wales, by Francis, Laycock, and Ratter (2019) among 99 Anglican clergy in England, by Francis, Crea, and Laycock (2021) among 287 priests in the Roman Catholic Church in Italy, and by Francis, Village, and Haley (2023) among 803 Methodist ministers in Britain.

While the Francis Burnout Inventory was originally developed for use in English-speaking communities, the Italian translation has now been well tested and employed in a series of studies (Francis & Crea, 2015, 2018, 2021; Francis, Crea, & Laycock, 2017, 2021; Francis, Laycock, & Crea, 2017). More recently, translations have been prepared and tested for use among clergy in Brazil (Fabri et al., 2025b) and in Portugal (Fabri et al., 2025a).

### Research Question

Against this background, the aim of the present study was: to translate the Francis Psychological Type and Emotional Temperament Scales into Portuguese; to generate and test short six-item measures to assess the orientations, attitudes, perceiving process, judging process, and emotionality; to test the predictive power of these short scales on the two scales proposed by the FBI (Scale of Emotional Exhaustion in Ministry and Satisfaction in Ministry Scale); and to compare the variance explained by these personality measures with the variance explained by lifestyle choices and professional support choices.

## Method

### Procedure

Before the study reported in the present paper, recognised procedures had been followed for translating the Francis Psychological Type and Emotional Temperament Scales for application in Portugal. The stages adopted were those universally recommended by Beaton et al. (2007) for cross-cultural adaptation and validation, namely assessment of conceptual and item equivalence; assessment of semantic and idiomatic equivalence; pretest of the final version; presentation of the translated and adapted version of the instrument to the authors; and content validation. For the present study, data were collected from a snowball sample of 266 Catholic priests serving in all regions of Portugal, including diocesan and religious priests. Data were collected using an anonymous online form hosted on the *LimeSurvey* Platform and sent by email or social media. Data were collected from February 2024 to July 2024.

### Participants

Of the 266 Catholic priests serving in Portugal who completed the measure of burnout, 208 also completed the measure of psychological type. Of these, 190 (91.3%) were diocesan priests and 18 (8.7%) religious priests; and 133 (63.9%) held postgraduate qualifications. In terms of age, 19.2% were under the age of forty, 32.2% were in their forties, 20.7% were in their fifties, 14.9% were in their sixties, and 13.0% were aged seventy or above.

### Measures

*Burnout* was assessed by the Portuguese translation of the two scales proposed by the Francis Burnout Inventory (Fabri et al., 2025a). This instrument comprises two 10-item scales. The 10-item Scale of Emotional Exhaustion in Ministry (SEEM) assesses negative affect. The 10-item Satisfaction in Ministry Scale (SIMS) assesses positive affect. Each item is rated on a five-point Likert scale: disagree strongly (1), disagree (2), not certain (3), agree (4), and agree strongly (5). Fabri et al. (2025b) reported the following Cronbach alphas for the two scales: SEEM, *α* = .89; SIMS, *α* = .89.

*Personality* was assessed by an experimental Portuguese translation of the 50 items proposed by the Francis Psychological Type and Emotional Temperament Scales (FPTETS: Village & Francis, 2023).

*Lifestyle choices* were assessed by the question, ‘How frequently do you do leisure activities?’ rated on a five-point scale: never (1), rarely (2), once a month (3), once a week (4), and more than once a week (5).

*Professional support choices* were assessed by the question, ‘Are you accompanied by a discipler or mentor?’ rated on a two-point scale: no (1), and yes (2).

### Analysis

The data were analysed by means of the SPSS software using the frequency, reliability, correlation, and regression routines.

## Results and Discussion

The first step in data analysis examined the frequency responses to the two items concerning lifestyle choices and professional support choices. The data presented in Table 1 demonstrate just over one quarter of the priests are accompanied by a discipler or mentor (26.4%) and that just over two fifths of the priests engage in leisure activities more than once a week (42.8%).

**Table 1:** Lifestyle choices and professional support choices

	%
*How frequently do you do leisure activities?*	
Never	1
Rarely	17
Once a month	7
Once a week	32
More than once a week	43
*Are you accompanied by a discipler or mentor?*	
No	73
Yes	27

N = 208

The second step in data analysis explored the scaling properties of the Portuguese translation of the FPTETS. Initial scaling analyses indicated that not all of the translated items functioned satisfactorily. Consequently, reliability analyses were employed to identify the best sets of six items within each of the five groups of items. Table 2 presents the alpha coefficient for each set of six items, the correlations between each item and the sum of the other five items in the set, and the percentage endorsement of the choices reflecting extraversion, sensing, thinking, judging, and emotional instability.

**Table 2:** FPTETS: Scale properties

			*r*	%
*Extraversion* (α = .70)				
Active	or	Reflective	.29	57
Sociable	or	Private	.65	52
Having many friends	or	A few deep friendships	.38	36
Like parties	or	Dislike parties	.42	63
Socially involved	or	Socially detached	.40	60
Talkative	or	Reserved	.49	51
*Sensing* (α = .62)				
Interested in facts	or	Interested in theories	.49	90
Practical	or	Inspirational	.35	68
The concrete	or	The abstract	.52	94
Prefer to make	or	Prefer to design	.45	77
Present realities	or	Future possibilities	.26	73
Down to earth	or	Up in the air	.19	88
*Thinking* (α = .63)				
Concerned for justice	or	Concerned for harmony	.31	40
Analytic	or	Sympathetic	.38	31
Tend to be firm	or	Tend to be gentle	.45	35
Logical	or	Humane	.33	19
Seek for truth	or	Seek for peace	.42	59
Fair-minded	or	Warm-hearted	.25	34
*Judging* (α = .68)				
Structured	or	Open-ended	.41	38
Orderly	or	Easy-going	.45	52
Organised	or	Spontaneous	.47	47
Punctual	or	Leisurely	.23	67
Like detailed planning	or	Dislike detailed planning	.45	64
Systematic	or	Casual	.49	66
*Instability* (α = .80)				
Discontented	or	Contented	.47	9
Feel insecure	or	Feel secure	.49	18
Have mood swings	or	Stay stable	.57	25
Get angry quickly	or	Remain placid	.55	24
Panic easily	or	Stay calm	.61	12
Frequently get irritated	or	Rarely get irritated	.68	24

N = 208

*r* = correlation between the individual items and the sum of the remaining items

% = per cent endorsement of items in the first column

The item endorsements generally indicated a stronger preference for extraversion over introversion among these priests, with more than half of them regarding themselves as preferring social involvement (60%), liking parties (63%), and being active (57%) rather than reflective. The item endorsements consistently indicated a stronger preference for sensing over intuition among these priests, with more than three quarters of them regarding themselves as preferring facts over theories (90%), seeing themselves as down to earth rather than up in the air (88%), and valorising the concrete over the abstract (94%). The item endorsement consistently indicated a stronger preference for feeling over thinking among these priests, with at least two-thirds of them preferring to be seen as humane (81%) rather than logical, as sympathetic (69%) rather than analytic, and as warm-hearted (66%) rather than fair-minded. The item endorsement generally indicated a preference for judging over perceiving among these priests, with around two-thirds of them seeing themselves as punctual (67%) rather than leisurely, as systematic (66%) rather than casual, and as preferring detailed planning (64%). The item endorsement consistently framed these priests as emotionally stable, with just 9% seeing themselves as discontented, 12% seeing themselves as someone who panics easily, and 18% recognising themselves as insecure.

Table 3 completes the description of the five six-item scales developed from the FPTETS by presenting the means and standard deviations. Table 3 also presents the mean scale scores for the Scale of Emotional Exhaustion in Ministry (*α* = .89) and for the Satisfaction in Ministry Scale (*α* = .89).

**Table 3:** Mean scale scores

	Means	SD
Extraversion	3.18	1.87
Sensing	4.90	1.32
Thinking	2.19	1.65
Judging	3.34	1.81
Instability	1.12	1.64
Emotional exhaustion	24.04	7.50
Satisfaction in ministry	40.92	5.37

N = 208

The third step in data analysis examines the bivariate correlations between scores recorded on SEEM and SIMS and personal factors (age and educational level), psychological factors (extraversion, sensing, thinking, judging, and emotionality), lifestyle factors (leisure activities), and professional factors (discipler or mentor). These bivariate correlations presented in Table 4 suggest that extraversion and emotionality are strongly correlated with both SEEM and SIMS in opposite directions. Additionally, sensing is negatively correlated with SEEM, and thinking is negatively correlated with SIMS. Age is negatively correlated with SEEM, but independent of SIMS. Leisure activities are strongly correlated with SEEM and SIMS in opposite directions. Additionally having a discipler or mentor is positively correlated with SIMS but independent of SEEM.

**Table 4:** Correlations with SEEM and SIMS

	SEEM *r*	SIMS *r*
*Personal factors*		
Age	−.18**	.08
Educational level	.01	.09
*Psychological factors*		
Extraversion	−.24***	.37***
Sensing	−.19**	.07
Thinking	.04	−.18**
Judging	.07	.04
Instability	.58***	−.46***
*Lifestyle factors*		
Leisure activities	−.33***	.27***
*Professional factors*		
Discipler/mentor	−.03	.19**

N = 208

**p < .01, ***p < .001

The fourth step is data analysis explores the bivariate correlations between the five personality variables and education, age, leisure activities, and having a mentor. The bivariate correlations presented in Table 5 suggest that extraverts are more likely than introverts to engage in leisure activities and to engage with a discipler or mentor; and that intuitive types are more likely to embrace higher levels of education and to engage with a discipler or mentor.

**Table 5:** Correlations with age, education, leisure activities, and professional support

	Education *r*	Age *r*	Leisure *r*	Mentor *r*
Extraversion	−.08	−.03	.20**	.18**
Sensing	−.23***	.15*	.04	−.16*
Thinking	−.04	.16*	.02	−.09
Judging	.13	.01	.04	.09
Instability	−.01	−.11	−.12	.02

*p < .05, **p < .01, ***p < .001

The final step in data analysis employs multiple regression to explore the effect on SEEM and SIMS separately of personal factors, psychological factors, lifestyle factors, and professional factors entered in that fixed order. In terms of SEEM, Table 6 demonstrates that the largest proportion of variance was accounted for by step 2, the psychological factors. When psychological factors were in the model, lifestyle factors explained additional variance, but professional factors did not. The beta weights in model 4 demonstrate that emotional exhaustion is higher among those who score highly on instability (*β* = .52), prefer introversion (*β* = −.13), and do not engage with leisure activities (*β* = −.25). Also emotional exhaustion is higher among younger priests (*β* = −.12). When all variables are in the model, neither the perceiving functions (sensing or intuition) nor the judging functions (thinking or feeling) explain additional variance in emotional exhaustion.

**Table 6:** Regression on SEEM

	*r*	Model 1 *β*	Model 2 *β*	Model 3 *β*	Model 4 *β*
*Personal factors*					
Age	−.18**	−.19**	−.12*	−.12*	−.12*
Educational level	.01	−.03	−.03	−.05	−.05
*Psychological factors*					
Extraversion	−.24***		−.19***	−.13*	−.13*
Sensing	−.19**		−.03	−.06	−.05
Thinking	.04		−.04	−.03	−.03
Judging	.07		.03	.05	.05
Instability	.58***		.55***	.52***	.52***
*Lifestyle factor*					
Leisure activities	−.33***			−.24***	−.25***
*Professional factor*					
Discipler/mentor	−.03				.01
R^2^		.034	.395	.451	.451
Δ		.034*	.362***	.055***	.000

*p < .05, **p < .01, ***p < .001

In terms of SIMS, Table 7 demonstrates that the largest proportion of variance was accounted for by step 2, the psychological factors. When psychological factors were in the model, lifestyle factors explained additional variance, but professional factors did not. The beta weights in model 4 demonstrate that satisfaction in ministry is higher among those who record low scores on instability (*β* = −.40), prefer extraversion (*β* = .29), and engage with leisure activities (*β* = .14). When all variables are in the model, neither the perceiving functions (sensing or intuition) nor the judging functions (thinking or feeling) explain additional variance in satisfaction in ministry.

**Table 7:** Regression on SIMS

	*r*	Model 1 *β*	Model2 *β*	Model 3 *β*	Model 4 *β*
*Personal factors*					
Age	.08	.11	.09	.09	.11
Educational level	.09	.12	.10	.11	.12*
*Psychological factors*					
Extraversion	.37***		.34***	.31***	.29***
Sensing	.07		−.06	−.05	−.03
Thinking	−.18**		−.08	−.09	−.09
Judging	.04		.12*	.10	.09
Instability	−.46***		−.42***	−.40***	−.40***
*Lifestyle factor*					
Leisure activities	.27***			.16**	.14*
*Professional factor*					
Discipler/mentor	.19**				.11
R^2^		.021	.350	.375	.387
Δ		.021	.330***	.025**	.011

*p < .05, **p < .01, ***p < .001

### Limitations

The first main limitation with the present study in that this is the first attempt to test a translation into Portuguese of the Francis Psychological Type and Emotional Temperament Scales. Although this translation did not support the recovery of the five ten-item scales, it permitted the creation of a satisfactory set of six-item scales. Further work is now needed to revise and test the translation of additional items. Meanwhile, however, these six-item scales have worked sufficiently well to provide data that can stand alongside and build on the more extensive work conducted and reported on the original English language version. The second main limitation with the present study is that this was not a longitudinal study, but a cross-sectional study. Consequently, the use of the term prediction in this study does not imply causal inference between independent and dependent variables.

## Conclusion

The present study was designed to test the predictive power of individual differences in personality (as defined and operationalised by the Francis Psychological Type and Emotional Temperament Scales) on susceptibility to burnout (as defined and operationalised by the Francis Burnout Inventory) among Catholic priests serving in Portugal, alongside the effect of engaging in leisure activities or engaging with a discipler or mentor. Data provided by 208 priests who completed the Francis Psychological Type and Emotional Temperament Scales, the Francis Burnout Inventory, and measures of leisure activity and engaging with a discipler or mentor lead to the following five main conclusions.

The first conclusion concerns the development of a short form of the Francis Psychological Type and Emotional Temperament Scales in Portuguese. The Portuguese translation worked relatively well. Satisfactory levels of internal consistency reliability were reported for the measures of stability–instability (*α* = .80) and introversion–extraversion (*α* = .70). The other three measures were less satisfactory and leave room for further refinement and development: sensing–intuition (*α* = .62), feeling–thinking (*α* = .63), and perceiving–judging (*α* = .68). Given that it takes time to nuance and refine measures of psychological type theory, the present instrument offers a good foundation on which to build.

The second conclusion concerns the psychological type profile of Catholic priests in Portugal as suggested by this new measure. The emerging picture is of a group of men who display more signs of extraversion than of introversion. They are much more concerned with a sensing (practical) approach to life than with an intuitive (imaginative) approach. They tend to focus on the present moment rather than to explore the future possibilities. They are more concerned with a feeling approach to life than with a thinking approach: they are more concerned for harmony than for justice. They are more concerned with a judging (structured) approach to the world than with a perceiving (flexible) approach. They display an emotionally stable profile rather than an unstable profile. Further work is needed to calibrate the FPTETS in Portugal to test these provisional findings.

The third conclusion concerns the power of personality theory to predict individual differences in scores recorded on the two scales proposed by the Francis Burnout Inventory. The most powerful personality factor is emotional instability: there is a strong positive path from emotional instability to emotional exhaustion in ministry (*β* = .58) and a strong negative path from emotional instability to satisfaction in ministry (*β* = −.40). The second most powerful personality factor is extraversion: there is a fairly strong positive path from extraversion to satisfaction in ministry (*β* = .29) and a less strong negative path from extraversion to emotional exhaustion in ministry (*β* = −.13). When both emotional instability and extraversion are taken into account, other personality factors are not significant.

The fourth conclusion concerns the role of leisure activities in predicting individual differences in burnout and the role of personality in predicting engagement with leisure activities. The data demonstrated that, although extraverts were more likely to engage with leisure activities, when emotional instability and extraversion were taken into account, engagement with leisure activity continued to provide additional predictive power on individual differences in susceptibility to burnout: there is a fairly strong negative path from leisure activities to emotional exhaustion in ministry (*β* = −.25) and a less strong positive path from leisure activities to satisfaction in ministry (*β* = .14).

The fifth conclusion concerns the role of engagement with a discipler or mentor in predicting individual differences in burnout and the role of personality in predicting engagement with a discipler or mentor. The data demonstrated that extraverts were more likely to engage with a discipler or mentor. However, when emotional instability and extraversion were taken into account, engagement with a discipler or mentor provided no additional predictive power on individual differences in susceptibility to burnout. In other words, while mentoring initially appears to help some priests experience higher levels of satisfaction in ministry, this apparent effect disappears because it is extraverts who are more likely to engage with a mentor.

There are two main implications from these findings for the pastoral care and pastoral oversight of Catholic priests in Portugal. The first implication is that the routine psychological profiling of seminarians preparing for priesthood could help to identify those more vulnerable to burnout in ministry. Such profiling should not be employed to exclude the more vulnerable but to support them. Self-awareness of vulnerability is itself a key protective factor. Moreover, the balanced affect approach to conceptualising professional burnout posits the beneficial effects of positive affect to ameliorate the detrimental effects of negative affect. The pastoral care of more vulnerable priests could, therefore, focus on enabling these priests to identify, recognise, and maximise the factors that nurture their positive affect.

The second implication from these findings for the pastoral care and pastoral oversight of Catholic priests in Portugal is that frequent engagement with leisure activities reduces susceptibility to burnout. Ministry itself can be an all-demanding and all-consuming way of life. These data provide a helpful and salutary reminder that leisure activities may resource ministry rather than detract from ministry. Such a reminder resonates with the example set by Jesus himself when he invited his immediate followers to step away from public engagement to get some rest (Mark 6: 31).

## Data Availability

Data are available from the corresponding author upon reasonable request.
